# Unusually High Affinity of the PLK Inhibitors RO3280 and GSK461364 to HSA and Its Possible Pharmacokinetic Implications

**DOI:** 10.1021/acs.molpharmaceut.2c00849

**Published:** 2023-02-22

**Authors:** Jesús Fernández-Sainz, Pedro J. Pacheco-Liñán, Consuelo Ripoll, Joaquín González-Fuentes, José Albaladejo, Iván Bravo, Andrés Garzón-Ruiz

**Affiliations:** † Departamento de Química Física, Facultad de Farmacia, Universidad de Castilla-La Mancha, Av. Dr. José María Sánchez Ibáñez, s/n, 02071 Albacete, Spain; ‡ Centro Regional de Investigaciones Biomédicas (CRIB), Unidad Asociada de Biomedicina (UCLM-CSIC), C/ Almansa, 14, 02008 Albacete, Spain; § Departamento de Química Física, Facultad de Ciencias y Tecnologías Químicas, Universidad de Castilla-La Mancha, Avenida Camilo José Cela, 10, 13071 Ciudad Real, Spain

**Keywords:** RO3280, GSK461364, drug−protein binding, human serum albumin, fluorescence spectroscopy

## Abstract

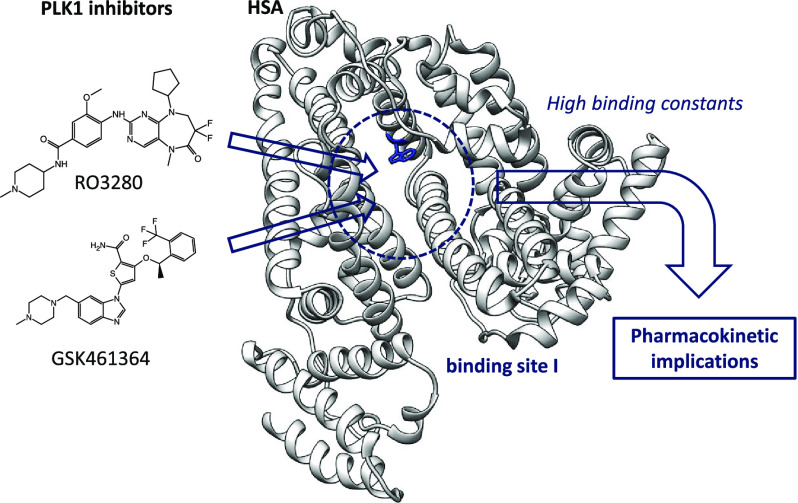

The binding processes of two Polo-like kinase inhibitors, RO3280 and GSK461364, to the human serum albumin (HSA) protein as well as the protonation equilibria of both compounds have been studied combining absorbance and fluorescence spectroscopy experiments together with density functional theory calculations. We found that the charge states of RO3280 and GSK461364 are +2 and +1, respectively, at the physiological pH. Nevertheless, RO3280 binds to HSA in the charge state +1 prior to a deprotonation pre-equilibrium. Binding constants to site I of HSA of 2.23 × 10^6^ and 8.80 × 10^4^ M^–1^ were determined for RO3280 and GSK461364, respectively, at 310 K. The binding processes of RO3280 and GSK461364 to HSA are entropy- and enthalpy-driven, respectively. The positive enthalpy found for the RO3280-HSA complex formation could be related to a proton pre-equilibrium of RO3280.

## 1 Introduction

**Figure 1:**
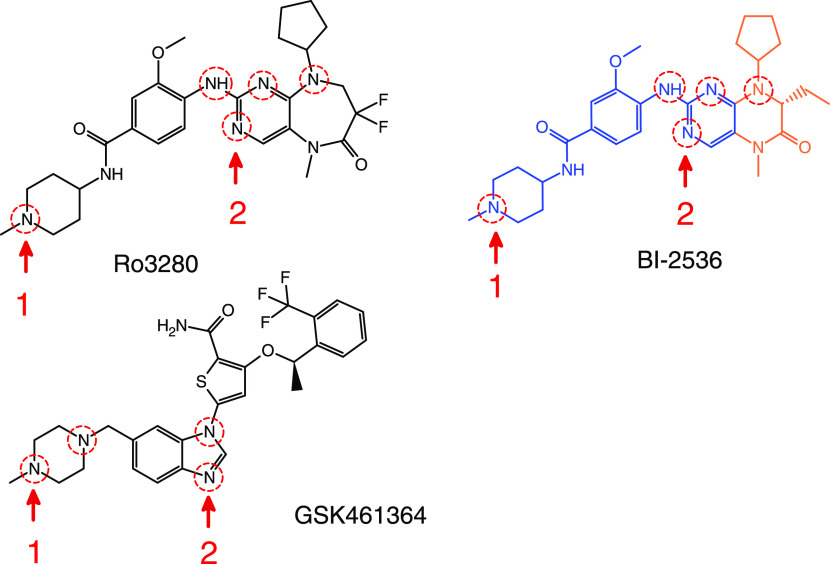
Chemical structures of RO3280 and GSK461364, along with the related inhibitor BI-2536. The blue color shows the chemical structure common to RO3280 and BI-2536. The orange color indicates the part of the molecule BI-2536 with high similarity to RO3280. The red dotted circles show the ionizable groups of the inhibitors, and the numbers 1 and 2 indicate the protonation order.

RO3280 and GSK461364 are selective ATP-competitive inhibitors of Polo-like kinase (PLK1).^1−3^ PLK1 belongs to a family of serine/threonine protein kinases, which plays a key role as a regulator of cell-cycle regulation.^4^ This protein is also involved in cancer progression and is overexpressed in malignant cells and solid tumors (lung, breast, ovary, stomach, colon).^5^ For this reason, PLK1 has emerged as an attractive target for cancer therapy.^3^ PLK1 inhibition results in the death of cancer cells by interfering with the cell cycle. This work is focused on the study of some physical properties of two novel PLK inhibitors, i.e., RO3280 and GSK461364 (see Figure 1). RO3280 is a pyrimidodiazepine-derived compound with an IC_50_ against PLK1 of 3 nM.^3,5^ This molecule exhibits high in vitro cellular potency in multiple leukemia cell lines and a significant level of antitumor activity in xenograft mouse models.^3^ Although RO3280 is still at the preclinical stage of development, it appears to have potential therapeutic value for acute myeloid leukemia inducing apoptosis and cell-cycle disorder.^6^ Its chemical structure has a high similarity to other PLK1 inhibitors such as BI-2536 and volasertib, which are currently in phase II of clinical trials. GSK461364, developed by Glaxo Smith Kline, is a selective thiophene amide inhibitor that displays at least 390-fold greater selectivity for PLK1 than for other PLK family proteins such as PLK2 and PLK3 and 1000-fold greater selectivity than for a panel of 48 other kinases.^2,7,8^ It also causes cell growth inhibition in a high percentage of tested cancer cell lines.^8^ Phase I clinical trials of GSK461364 in patients with advanced solid malignancies were reported in 2011.^2^

The pharmacokinetic profile of a drug closely depends on physicochemical parameters such as water solubility, lipophobicity, and affinity to blood proteins, mainly albumin, among others. Human serum albumin (HSA) plays a crucial role as a carrier of poor water-solubility drugs.^9^ Drug–albumin interaction has a direct effect on distribution, metabolism, and excretion, so the drug affinity to HSA must be considered in the pharmacokinetic and dose-finding studies. A high affinity to the albumin protein is typically associated with a low free drug concentration in blood. On the other hand, a high affinity to this protein can lead to extending the drug half-life^10^ and providing selective delivery in inflamed tissues and tumors.^11^ Thus, HSA nanoparticles have also been used as drug carriers for some antitumor drugs such as paclitaxel (Abraxane).^12^ In this work, a combination of fluorescence spectroscopy experiments and density functional theory (DFT) calculations was used to investigate two features related to the pharmacokinetics of RO3280 and GSK461364. First, we analyzed the protonation equilibria of both compounds and determined their p*K*_a_ and charge state in physiological conditions. These physical properties are closely related to the drug solubility and potential side effects. Second, the affinity and binding mechanism to HSA were evaluated and correlated with the half-life reported for different kinase inhibitors.

## 2 Materials and Methods

### 2.1 Chemicals

RO3280 (≥99.5%) and GSK461364 (≥99.8%) were supplied by MedChem Express. HSA (≥99%; fatty acid- and globulin-free), ibuprofen (>98%), warfarin (99.9%), Bis-Tris (≥98.0%), NaCl (≥99.0%), and DMSO (99.9%) were purchased from Sigma-Aldrich. The samples were dissolved in 0.02 M Tris-HCl buffer solutions at pH 7.4 containing 0.1 M NaCl. All of the buffer solutions were prepared in Milli Q water and filtered with 0.22 μm filters before being used.

### 2.2 Spectroscopy Experiments

The UV–vis absorption spectra of RO3280 and GSK461364 were acquired using a Cary 100 (Varian) spectrophotometer in a 10 mm quartz cuvette, with a step of 1 nm, scan rate of 600 nm min^–1^, and at room temperature. Steady-state fluorescence spectra were recorded employing two different spectrofluorometers: (1) the FLS920 (Edinburgh Instruments) spectrofluorometer equipped with a 450 W Xe lamp as the excitation source, an microchannel plate-photomultiplier tube (MCP-PMT) detector (R3809 model), and a time-correlated single photon counting (TCSPC) data acquisition card (TCC900 model); and (2) the FS5 spectrofluorometer (Edinburgh Instruments) equipped with an integrating sphere, a 150 W Xe lamp as the light source, and a PMT (photomultiplier tube) detector (R928P model). Synchronous fluorescence spectra were carried out in the FS5 spectrofluorometer at room temperature. A subnanosecond pulsed light-emitting diode, EPLED-290 (Edinburgh Photonics), was employed as the light source at 291 nm to acquire time-resolved fluorescence spectra with the FLS920 spectrofluorometer. The aperture of the slits (Δλ_ex_ and Δλ_em_) was chosen depending on the experiment. The step and dwell time were 1 nm and 0.1 s, respectively. A TLC 50 temperature-controlled cuvette holder (Quantum Northwest) was used for all of the fluorescence experiments.

For the spectroscopic characterization of the drugs, 10 μM solutions of RO3280 and GSK461364 were prepared in different organic solvents. Small volumes of concentrated HCl and NaOH solutions were added to the aqueous solutions of RO3280 and GSK461364 (10 μM) for pH titration experiments. Working solutions of HSA (5 μM, 3 mL) were prepared daily in Bis-Tris 0.02 M/NaCl 0.1 M buffer and titrated in a cuvette by small volumes of concentrated ethanolic solutions of RO3280 (10 mM) and GSK461364 (2 mM) for binding to protein experiments. The final concentrations of RO3280 and GSK461364 in the HSA solution varied from 0.0 to 50.0 μM (the [drug]:[HSA] ratios were 0, 1, 2, 3, 5, 7, and 10). For steady-state fluorescence spectra, the excitation wavelength chosen was 295 nm to excite the tryptophan residue Trp214 and to avoid the excitation of tyrosines. The emission fluorescence intensity was collected at 320 nm to avoid the fluorescence emission of the drugs. Inner filter effects were corrected through
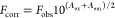
1where *F*_corr_ and *F*_obs_ are the corrected and observed fluorescence intensities, respectively, and *A*_ex_ and *A*_em_ are the absorbances at excitation and emission wavelengths (295 and 320 nm, respectively).^13^ The excitation and emission slits were fixed at 1 and 5 nm, respectively. The step and dwell times were 1 nm and 0.1 s, respectively. Binding to HSA experiments was performed at three different temperatures, i.e., 298, 303, and 310 K. The experiments were repeated at least six times for each temperature.

Time-resolved fluorescence emission was also collected at 320 nm. The fluorescence intensity decay, *I*(*t*), was fitted to the following multiexponential function using an iterative least-square fit method
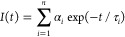
2where α*_i_* and τ*_i_* are the amplitude and lifetime, respectively, for each *i*^th^ term. The mean lifetime of the decay was then calculated as
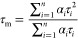
3

### 2.3 Computational Details

DFT calculations were carried out for different charge states of RO3280 and GSK461364. A previous conformational analysis was performed for the neutral state of both drugs in the gas phase to obtain the lowest energy conformation. The nature of the stationary points was assessed by means of normal vibration frequencies calculated from the analytical second derivatives of the energy. The PBE0 method^14^ as implemented in Gaussian16 (revision C.01),^15^ along with the 6–31G* and 6–31+G** basis sets, was used for the conformational analysis and the subsequent optimization of the molecular structure of the drugs in the neutral and charged states. The 6–31+G** basis set is especially recommended in calculations involving anionic species.^16^ The polarizable continuum model (PCM) was employed to include the solvent (water) effect.^17,18^

The electronic vertical transitions were calculated at the time-dependent (TD)-PBE0/6–31+G** level (including solvent effects). TD-PBE0 has previously been successfully employed to calculate low-energy transitions for BI-2636^19^ and other π-conjugated organic compounds.^20,21^

## 3 Results and Discussion

### 3.1 Spectroscopic Characterization and Protonation Equilibria

**Figure 2:**
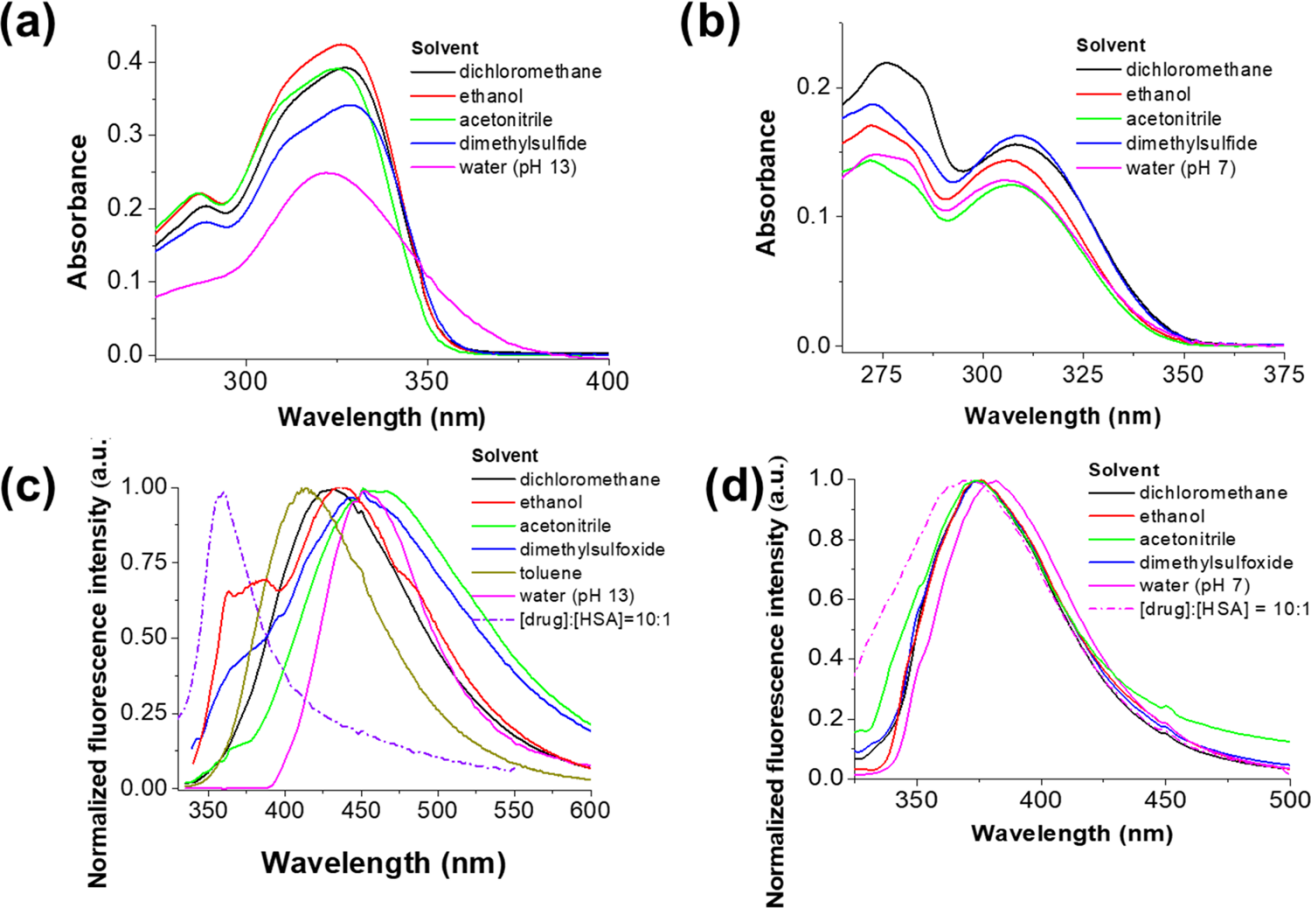
UV–vis absorbance spectra of (a) RO3280 and (b) GSK461364 in different solvents. Normalized emission spectra of (c) RO3280 and (d) GSK461364 in different solvents. The sample concentration was 10 μM.

The spectroscopic properties of the studied drugs were investigated in different solvents. Figure 2 shows the UV–vis absorption and fluorescence emission spectra acquired for both inhibitors (the maximum absorption and emission wavelengths are collected in Table S1). The absorption spectra were not significantly sensitive to solvent polarity, while the emission band of RO3280 undergoes a bathochromic shift in polar solvents due to an intramolecular charge transfer (ICT) process in the excited state as previously reported for the related inhibitor BI-2536.^19^

**Figure 3:**
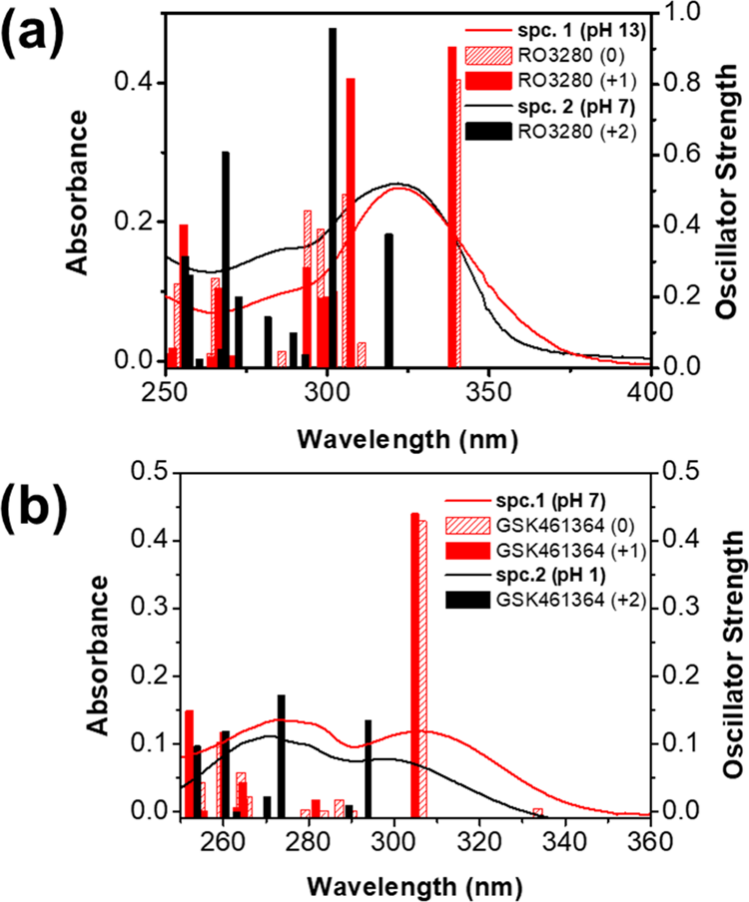
Absorption spectra of (a) RO3280 and (b) GSK461364 at different pH values (the sample concentration was 10 μM). The vertical bars correspond to the oscillator strengths (*f*) calculated for the electronic transitions of RO3280 and GSK461364 in different charge states, at the TD-PBE0/6–31+G** level of theory. Calculated electronic transitions indicate that the charge states 0 and +1 are spectroscopically indistinguishable and both were considered spectroscopic species 1 (spc.1). Spectroscopic species 2 (spc.2) correspond to the charge state +2.

**Figure 4:**
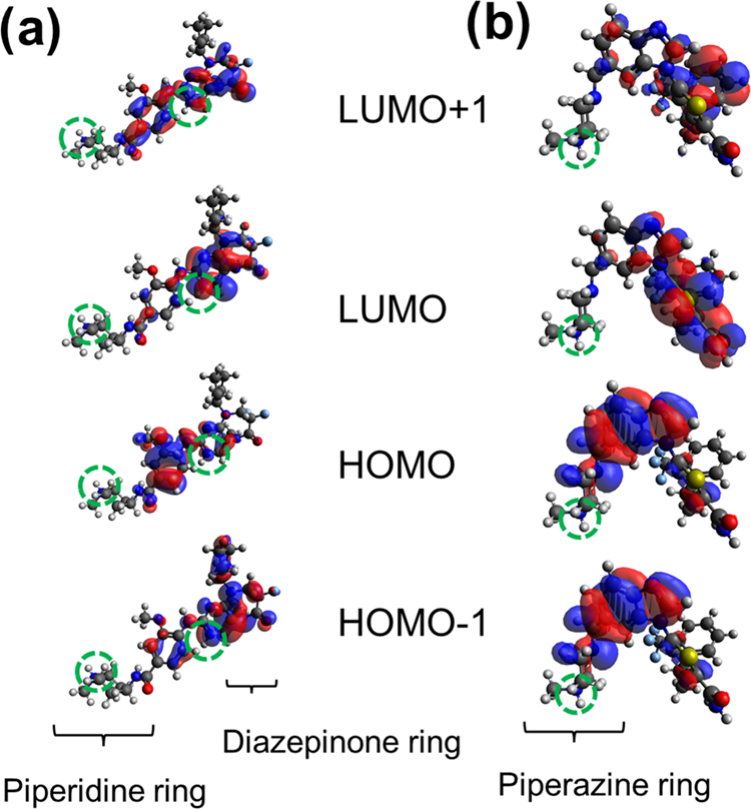
Frontier molecular orbital calculated for RO3280 in the diprotonated form and GSK461364 in the monoprotonated form. Green dotted cycles indicate the ionized groups at the physiological pH.

In aqueous solutions, the spectroscopic properties of the studied inhibitors exhibit some interesting differences with respect to those observed in organic solvents. The ionization state of the inhibitor has a strong impact on absorption and emission properties. This dependence was employed to determine the most abundant charge state of each inhibitor at the physiological pH. The charge state of a drug has particular relevance in physicochemical properties such as solubility, lipophobicity, and affinity to HSA and its target proteins.^22^ RO3280 and GSK461364 present different ionizable groups (secondary and tertiary amines) highlighted with dotted circles in Figure 1. The chemical structure of RO3280 shows high similarity to BI-2535, and both molecules have the same amine groups with comparable chemical environments. Accordingly, RO3280 was optimized in the neutral form and charge states +1 and +2 at the PBE0/6–31+G** level of theory, assuming the same protonation order as that reported for BI-2535 (see Figure S4).^19^ Close wavelengths (differences ≤ 2 nm) were computed for the lowest-energy vertical transitions of BI-2535 with charges 0 and +1, suggesting that the neutral and monoprotonated forms are hardly distinguishable by UV–vis absorption spectroscopy (see Figure 3a and Table 1). This is a reasonable result because the molecular orbitals involved in these vertical transitions are not localized on the piperidine ring, where the first protonation equilibrium occurs. Therefore, in Table 1 and Figure 3a, the absorption band of the spectroscopic species 1 was assigned at both the neutral state of RO3280 and its monoprotonated form (charge states 0 and +1). An isosbestic point at 340 nm and a p*K*_a_ of 12.6 was found for the protonation equilibrium between the spectroscopic species 1 and 2 (see Figure S1). Species 2 is predominant at the physiological pH and was assigned to the charge state +2 as previously reported for BI-2535.^19^ TD-PBE0/6–31+G** calculations showed a good match between the wavelengths calculated for the transitions S_0_ → S_1_ and S_0_ → S_2_ of the diprotonated form of RO3280 (λ_vert_^calc^ = 319 and 302 nm, respectively) and the experimental absorption maximum determined at pH 7.4 (λ_ab_^max^ = 324 nm). As shown in Figure 4, HOMO and LUMO have a different electronic density distribution, and this fact can be associated with the previously mentioned ICT process. At acidic pH values, a new absorption spectrum appears, which can be associated with a species with charge +3 (λ_ab_^max^ = 304 nm; see Figure S1), as previously reported for BI-2535.^19^ An isosbestic point at 306 nm and a p*K*_a_ of 3.8 was found for that protonation equilibrium. We did not delve into the spectroscopic characteristics of this species because of its little relevance at physiological conditions. RO3280 does not exhibit fluorescence in aqueous solutions at the physiological pH, while an emission band centered at 452 nm appears at pH ≥ 13. In the charge state +2, the protonation at position 2 (involving the electron lone pair of the nitrogen atom) results in a loss of electron density and a quenching of the fluorescence signal, while the neutral and monoprotonated forms (pH ≥ 13) have an emission maximum close to those recorded in polar solvents such as dimethylsulfoxide and acetonitrile (451 and 462 nm, respectively) (see Figure 1 and Table S1).

**Table 1:** Lowest-Energy Absorption Maximum (λ_ab_^max,1^) Recorded for RO3280 and GSK461364 in Different pH Ranges^a^

inhibitor	pH	λ_ab_^max,1^ (nm)	charge state	transition	λ_vert_^calc^ (nm)	*f*	main component of the transition (>10% contribution)
RO3280	≥13	322	0	S_0_ → S_1_	340	0.405	H-1 → L (62%); H → L (23%)
				S_0_ → S_3_	306	0.240	H-2 → L (59%); H-1 → L (15%)
	≥13	322	+1	S_0_ → S_1_	338	0.452	H → L (85%); H-1 → L (11%)
				S_0_ → S_2_	307	0.406	H-1 → L (55%); H → L + 1 (31%)
	5–13	321	+2	S_0_ → S_1_	319	0.183	H → L (93%)
				S_0_ → S_2_	302	0.479	H → L + 1 (69%)
GSK461364	≥3	308	0	S_0_ → S_2_	306	0.429	H-1 → L (95%)
	≥3	308	+1	S_0_ → S_1_	305	0.440	H→ L (97%)
	≤1	298	+2	S_0_ → S_1_	294	0.135	H→ L (47%); H-1 → L (47%)

a Vertical transition wavelengths (λ_vert_^calc^) calculated for RO3280 and GSK461364 in different charge states along with the oscillator strength (*f*) and the main component of the transition.

At physiological pH, the absorption spectrum of GSK461364 is similar to those recorded in polar organic solvents (see Figure 1 and Table S1). The electronic vertical transitions of GSK461364 were calculated in charge states 0, +1, and +2 to interpret the spectroscopic observations. The protonation order was previously computed because the molecular structure of GSK461364 has large differences with respect to RO3280 and BI-2535. Δ*G*^0^ was calculated for different protonation equilibria of the drug considering a free energy of −270.28 kcal mol^–1^ for the proton in aqueous solutions according to the recommendation of Camaioni and Schwerdtfeger.^23^ A scheme with all of the studied protonation equilibria for GSK461364 is shown in Figure S5. As expected, the first ionized group corresponds to one of the amine groups of the terminal piperazine ring (an auxophore moiety). The neutral and monoprotonated forms of GSK461364 cannot be easily distinguished by UV–vis absorption spectroscopy since close wavelengths were computed for the lowest-energy vertical transitions of both charge states (see Figure 3b and Table 1). This is because the ionized amine group does not contribute to molecular orbitals involved in these transitions (Figure 4). Consequently, both the neutral and monoprotonated forms (charge states 0 and +1) were assigned to the spectroscopic specie 1. Nevertheless, the monoprotonated form of GSK461364 should be predominant at the physiological pH since a p*K*_a_ of 8.38 has been reported for the 1,4-dimethylpiperazine molecule at 298 K.^24^ In contrast to RO3280 and BI-2535, GSK461364 exhibits fluorescence, with an emission maximum at 380 nm, at the physiological pH since the protonation of the piperazine ring does not have a significant effect on the conjugated part of the molecule (Figure S3 and Table S1). At strongly acidic conditions, a new spectral band appears, with an isosbestic point at 273 nm and a p*K*_a_ value of 1.4 (Figures 3 and S2). This new species (named spectroscopic species 2) was assigned to the diprotonated form of GSK461364. DFT calculations predicted that the second protonated group corresponds to the secondary amine of the benzimidazole moiety (Figure S5).

### 3.2 Protein Binding Experiments

**Figure 5:**
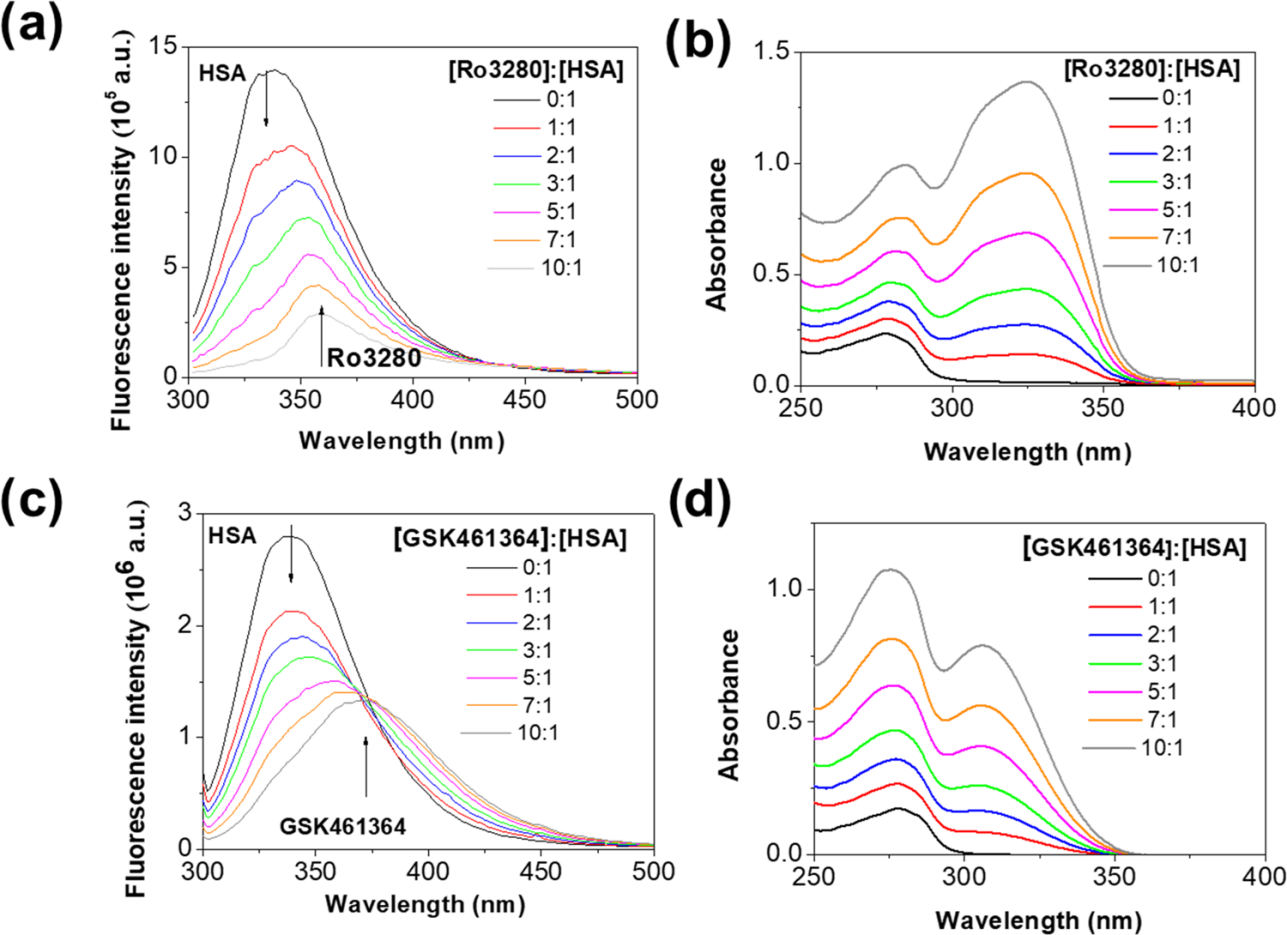
Effect of (a) RO3280 and (c) GSK461364 on the fluorescence spectrum of HSA (λ_ex_ 295 nm, Δλ_ex_ 1 nm, Δλ_em_ 5 nm, step 1 nm, dwell time 0.1 s; T 310 K; protein concentration was 5 μM). (b) and (d) are the absorption spectra recorded in experiments (a) and (c).

HSA solutions (in Tris-HCl buffer at pH 7.4) were titrated with the studied drugs, and in both cases, it was observed a strong decrease of the fluorescence intensity of the protein (see Figure 5a,c). Such strong fluorescence HSA quenching is generally associated with interactions with the single tryptophan residue (Trp214) at the distal end of the site 1 pocket.^25^ It is well known that this protein has two main binding sites.^26−29^ Site 1 located in subdomain IIA is mainly a nonpolar binding site formed by a hydrophobic pocket. This site has a high affinity to drugs with an aromatic structure, a lipophilic character, or surrounded by negative charges such as warfarin, salicylic acid, amantadine, or iodipamide.^26,29,30^ Site 2 (in subdomain IIIA) is smaller and more stereoselective than site 1, and drugs such as diazepam, paracetamol, ibuprofen, and propofol, among others, are attached to this site.^29,31^ We confirmed the higher affinity of RO3280 and GSK461364 to site 1 by means of competitive experiments with warfarin and ibuprofen as described below. In the protein–ligand titration experiments, a bathochromic shift of the fluorescence emission maximum (in addition to the fluorescence intensity quenching) was observed because of the emission signal of the drug. The emission maxima observed at the [GSK461364]:[HSA] ratios of 10:1 (375 nm) and 25:1 (380 nm) are comparable to the maximum recorded for GSK461364 in aqueous solutions (380 nm) (Figures 5c and S3). The case of RO3280 is more complex because this molecule does not exhibit fluorescence at charge state +2, predominant at the working pH. Hence, the presence of an emission maximum at 360 nm for the [RO3280]:[HSA] ratio of 10:1 (Figures 2c and 5a) suggests that the inhibitor loses the proton at position 2 in a pre-equilibrium before protein binding, as reported for the binding of BI-2536 to HSA and PLK1.^19^ Interestingly, the emission band of RO3280 in the presence of HSA is significantly hypsochromically shifted with respect to the emission maximum recorded in polar organic solvents and aqueous solutions at pH 13. This feature can be attributed to the sensitivity of the emissive properties of RO3280 to the polarity of the medium, suggesting that the ligand is located in a hydrophobic pocket of the protein. Figure 5b,c shows the absorption spectrum of HSA in absence and presence of different concentrations of inhibitors. The band centered at about 280 nm is commonly attributed to π–π* transitions of aromatic amino acid residues in HSA. Unfortunately, the changes in the secondary structure of the protein cannot be analyzed because of the strong absorbance of both inhibitors between 250 and 350 nm (Figures 2a,b and 3). For instance, GSK461364 has an absorption band centered at 275 nm at pH 7.0. In the presence of HSA, that band shows a higher absorbance than the band at 308 nm due to the contribution of the protein (Figure 5d). As shown, no significant spectral shifts were observed for the band centered at about 280 nm.

**Figure 6:**
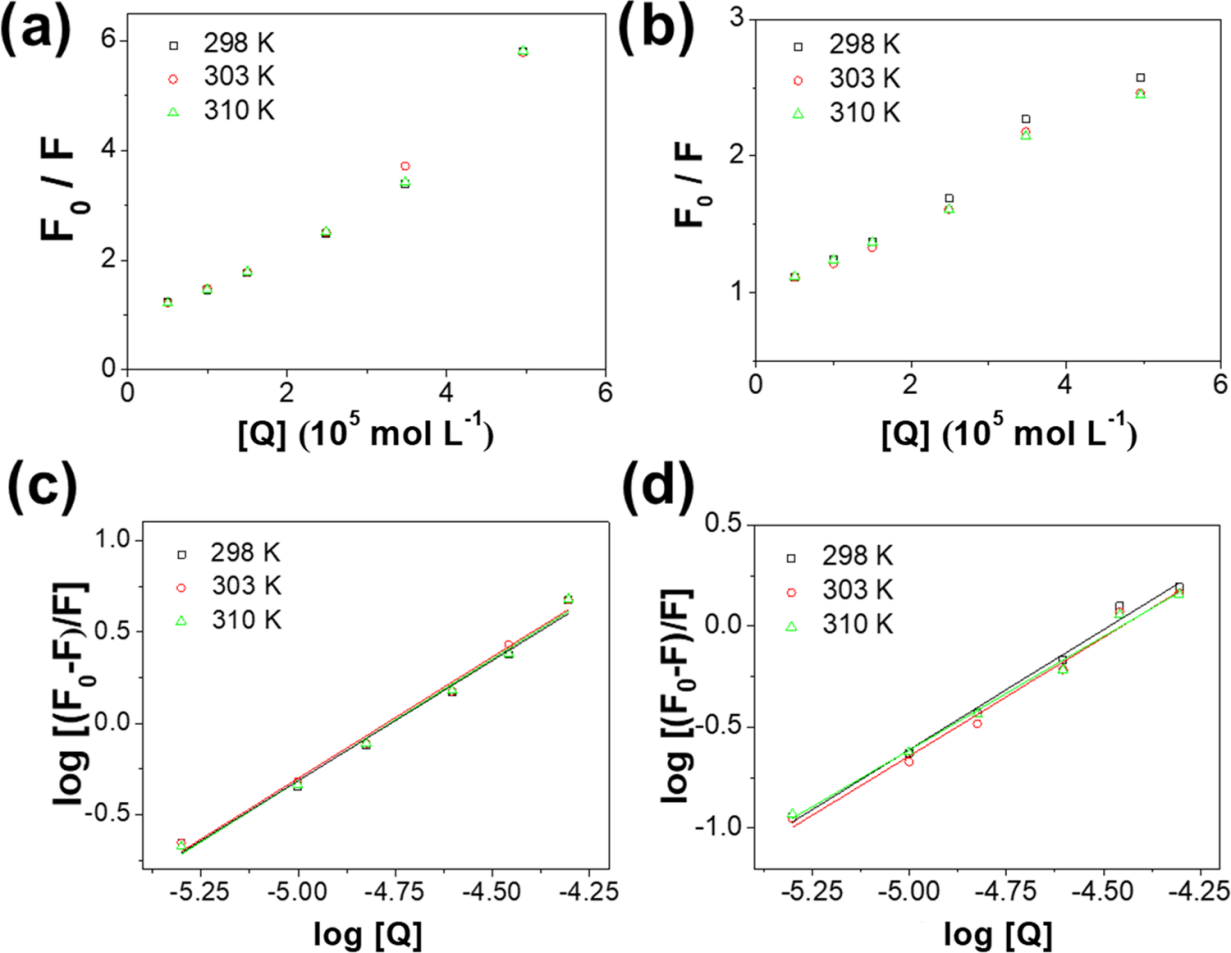
Stern–Volmer plots for the binding of (a) RO3280 and (b) GSK461364 to HSA. Plot of log [(*F*_0_ – *F*)/F] vs log [*Q*] of (c) RO3280 and (d) GSK461364. Experiments were performed at an HSA concentration of 5 μM and different temperatures.

An upward curvature was observed in the Stern–Volmer plots of both ligands (Figure 6a,b). This fact indicates that static quenching plays a significant role in the deactivation process of the excited state of the protein. Thus, the classic Stern–Volmer equation can be reformulated when the relative fluorescence intensity becomes the product of dynamic and static quenching contributions as
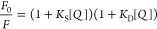
4where *K*_S_ and *K*_D_ are the static and dynamic quenching constants, respectively.^13,32^ In addition, no significant variations of the protein fluorescence lifetime were observed in the presence of small [drug]:[protein] ratios for both GSK461364 and RO3280 (see Table S2). This fact suggests that the drug binding to the protein is the main mechanism contributing to fluorescence deactivation since the fluorescence lifetime is not significantly affected by static quenching.^13^ The same effect has been observed for different fluorescence quenchers of serum albumins such as isoflavones, phenols, and flavonoids.^33−35^

The binding constant to HSA was obtained according to the following equation
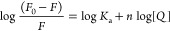
5where *n* and *K*_a_ are the number of binding sites and the binding constant, respectively (see Figure 6c,d).^21^Table 2 summarizes the binding parameters determined for RO3280 and GSK461364 obtained at different temperatures along with the previously reported data for BI-2536. The high binding constants obtained for the studied drugs, and particularly for RO3280, indicate a strong affinity to HSA. The constants determined for RO3280 are comparable to those reported for BI-2536.^19^ At 310 K, for instance, the binding constants of RO3280 and BI-2536 are 2.23 × 10^6^ and 3.78 × 10^6^ M^–1^, respectively, with the constant determined for GSK461364 (8.80 × 10^4^) being 25 times lower than for RO3280. This is a reasonable result considering the structural similarity existing between RO3280 and BI-2536. Smaller binding constants to HSA are generally reported for more conventional drugs such as furosemide (1.99 × 10^5^ M^–1^ at 310 K),^36^ axitinib (9.7 × 10^4^ M^–1^ at 308 K),^37^ tenofovir (5.7 × 10^4^ M^–1^ at 310 K),^38^ and lamotrigine (3.45 × 10^2^ M^–1^ at 308 K).^39^ The high affinity to HSA of RO3280 and BI-2536 with respect to other kinase inhibitors is revealed in Table 3. Most of the binding constants collected in the table are within the range 3.4 × 10^4^ M^–1^ (crizotinib) to 7.2 × 10^3^ M^–1^ (ibrutinib). The binding constant of GSK461364 can also be considered as high in comparison with those shown in Table 3, only exceeded by the constants of BI-2536, RO3280, and neratinib (2.25 × 10^5^ M^–1^).

**Table 2:** Binding and Thermodynamic Parameters from the HSA/Drug Complex

drug	*T* (K)	*K*_a_^c^ (10^6^ M^–1^)	*n*^c^ ± 2σ	Δ*H*^0^ (kJ mol^–1^)	Δ*S*^0^ (J K^–1^ mol^–1^)	Δ*G*^0^ (kJ mol^–1^)
RO3280	310	2.23	1.33 ± 0.05			–37.62
	303	1.65	1.30 ± 0.07	26.74	207.6	–36.17
	298	1.47	1.29 ± 0.06			–35.13
GSK461364	310	0.0880	1.11 ± 0.05			–29.20
	303	0.121	1.15 ± 0.03	–55.61	–85.20	–29.80
	298	0.208	1.19 ± 0.06			–30.22
BI-2536^a^	310	3.78	1.35 ± 0.16			–39.2
	303	1.86	1.30 ± 0.09	103.8	461.3	–36.0
	298	0.75	1.22 ± 0.16			–33.6

a Ref (19).

**Table 3:** Binding Constants to HSA or BSA Determined for Different Kinase Inhibitors at 310 K (or Near Temperatures). Binding Constants Were Obtained Both Including Inner Filter Corrections (*K*_a_^c^), according to eq 1, and Without These Corrections (*K*_a_^u^) for Comparative Purposes^a^

drug	protein	*T* (K)	*K*_a_^c^ (M^–1^)	*K*_a_^u^ (M^–1^)	Δ*H*^0^ (kJ mol^–1^)	Δ*S*^0^ (J K^–1^ mol^–1^)	Δ*G*^0^ (kJ mol^–1^)
BI-2536^b^	HSA	310	3.78 × 10^6^	11.4 × 10^7^	103.8	461.3	–39.2
Ro3280	HSA	310	2.23 × 10^6^	3.59 × 10^6^	26.74	207.6	–37.62
Neratinib^c^	HSA	310	2.25 × 10^5^				
GSK461364	HSA	310	8.80 × 10^4^	5.60 × 10^5^	–55.61	–85.20	–29.20
Crizotinib^d^	BSA	309	3.43 × 10^4^		–3.23	76.37	–26.83
Nazartinib (EGF816)^e^	HSA	310	2.67 × 10^4^		–3.22	74.36	–26.27
Sorafenib^f^	HSA	305	2.64 × 10^4^		–28.2	–7.87	–25.8
MK-0457^g^	HSA	310	1.05 × 10^4^		–130.63	–343.89	–23.02
Vandetanib^h^	HSA	303	7.63 × 10^3^		–6.57	52.76	–22.56
Ibrutinib^i^	HSA	310	7.2 × 10^3^		–16.91	26.44	–25.09
Linifanib^j^	BSA	308	4.3 × 10^3^		–55.91	–111.74	–21.49
Nilotinib^k^	HSA	310		1.07 × 10^3^	–38.76	–75.72	–17.99
Erlotinib^l^	BSA	307	3.44 × 10^2^		–260.80	–793.31	–17.26

a Thermodynamic parameters of the protein–drug binding.

b Ref (19).

c Ref (51).

d Ref (52).

e Ref (53).

f Ref (54).

g Ref (55).

h Ref (56).

i Ref (57).

j Ref (58).

k Ref (59).

l Ref (60)

Such high binding constants to HSA of the studied drugs should have an effect on their pharmacokinetic parameters. In this sense, four kinase inhibitors were selected from Table 3 to try to establish a correlation between the binding constants to HSA and the pharmacokinetic behavior. GSK461364, BI-2536, MK-0457, and nazartinib (EGF816) were chosen because (i) a binding constant to HSA at 310 K has been reported using a similar experimental procedure as in the present work, including inner filter corrections; and (ii) pharmacokinetic studies in phase I clinical trials have been performed. Unfortunately, no pharmacokinetic information is available for RO3280 because only preclinical studies have been reported to date. A high variability of pharmacokinetic profiles was found in the reported studies, which combine different dosage schedules and pathological conditions.^2,40−45^ Accordingly, it was not possible to establish a clear correlation between the binding constant to HSA and pharmacokinetic parameters. Nevertheless, the highest half-life in blood was reported for the compound with the highest binding constant to HSA (BI-2536) within the set of chosen kinase inhibitors. For instance, a terminal half-life of 15.4 h has been reported for a single intravenous infusion of BI-2536 (200 mg over 1 h).^44^ Half-life values around 25 hours (min 18.6, max 55.1) were found in a noncompartmental pharmacokinetic analysis of BI-2536 (administered as a 1 hour infusion at doses ranging from 25 to 200 mg on day 1 and day 8 of a 21 day treatment course).^40^ Similar results (terminal half-life up to 20–30 h) were reported when administered a 1 hour intravenous infusion of three doses (50, 60, and 70 mg) on days 1 and 3 of a 21 day treatment course.^43^ The largest volumes of distribution were also found for BI-2536 (751–2200 L), suggesting an extensive distribution into deeper compartments.^2,41,43^ Although a correlation among the binding constant to HSA, the half-life in blood, and the volume of distribution cannot be fully established, these results encourage further studies on RO3280, including clinical trials. RO3280 and BI-2536 have a similar molecular structure and binding constant to HSA, and a comparable pharmacokinetic profile could be expected. Additionally, it has been suggested that HSA could act as a reservoir of some drugs with high affinity to the protein.^46^ Thus, drugs with high affinity to HSA could be preferentially delivered to the tumor when HSA–ligand complexes were taken up and metabolized in it.^46−48^ This behavior could be explained by the highly permeable vasculature and insufficient lymphatic drainage observed in solid tumors, which leads to the accumulation of macromolecules (>40 kDa), preferentially HSA, in the tumor interstitium.^27,46,48,49^ This phenomenon is known as enhanced permeability and retention effect and could favor the selective transport of compounds with high affinity to the HSA such as RO3280 and BI-2536.^50^

**Figure 7:**
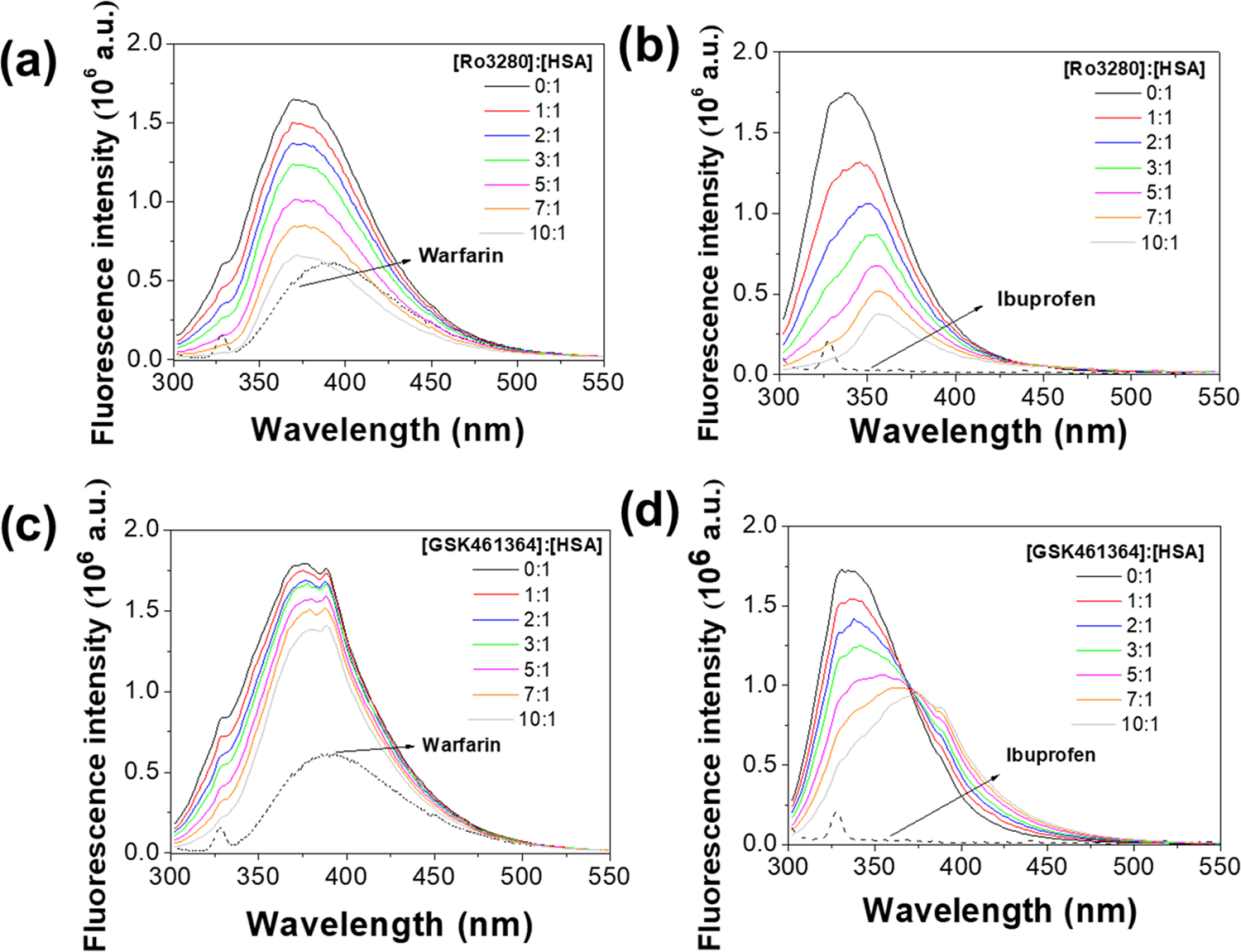
Competitive binding studies of RO3280 with (a) warfarin and (b) ibuprofen and GSK461364 with (c) warfarin and (d) ibuprofen (*T* = 310 K, λ_ex_ = 295 nm, [HSA] = [competitive drug] = 5 μM).

**Figure 8:**
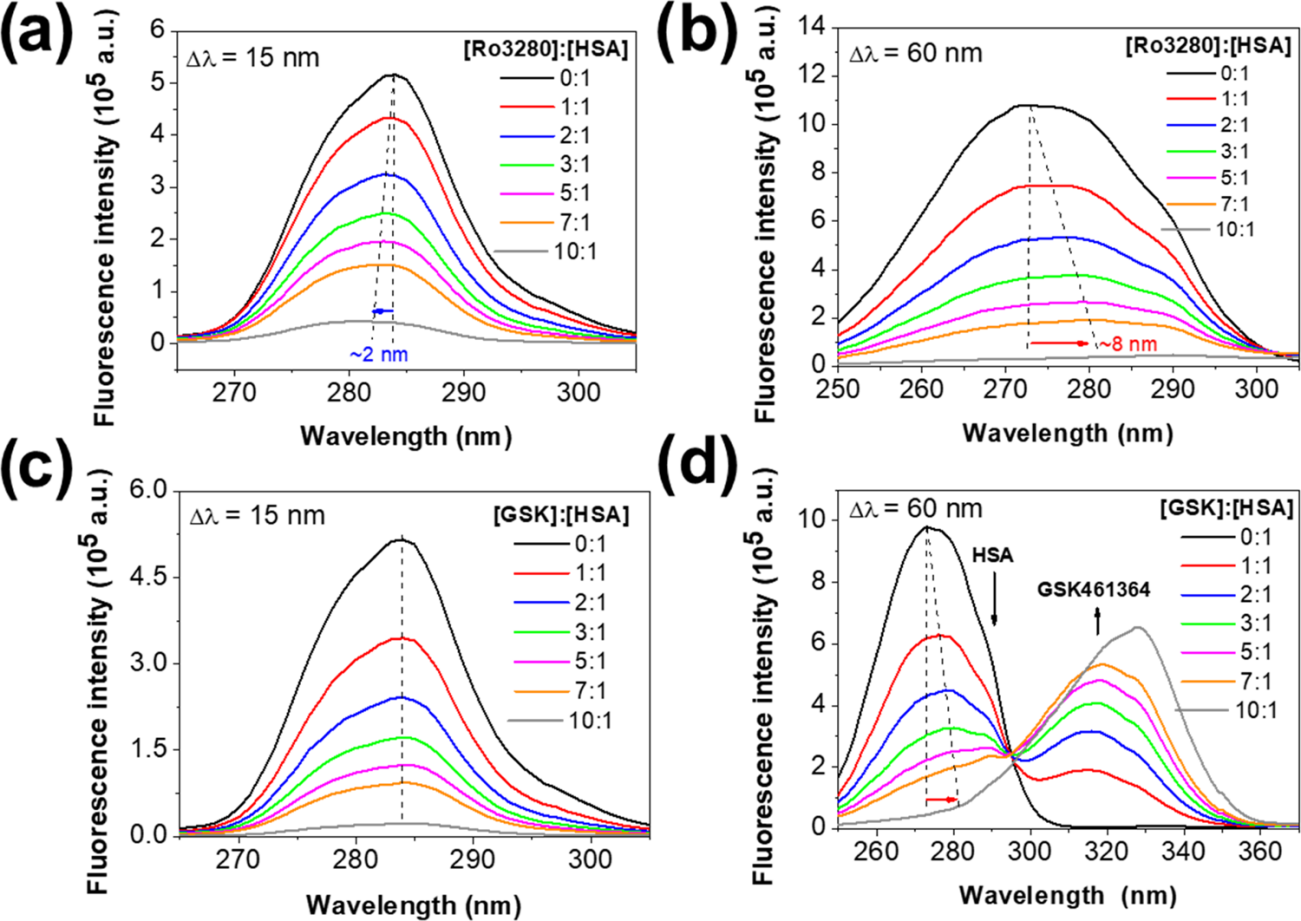
Effect of (a, b) RO3280 and (c, d) GSK461364 on the synchronous fluorescence spectra of HSA (Δλ = 15 nm for (a, c); Δλ = 60 nm for (b, d); protein concentration was 5 μM).

Competitive binding experiments with warfarin (site 1 binder) and ibuprofen (site 2 binder) were carried out to test the specific binding site of RO3280 and GSK461364 into HSA (see Figure 7). The HSA binding experiments were performed at 310 K using [HSA]:[warfarin] and [HSA]:[ibuprofen] ratios of 1. In these conditions, the binding constants determined for RO3280 (1.98 × 10^6^ M^–1^) and GSK461364 (8.95 × 10^5^ M^–1^) in the presence of ibuprofen did not show a significant variation from those obtained in its absence. On the contrary, the presence of warfarin led to a strong drop of the binding constant of the studied drugs, i.e., (4.42 × 10^4^ M^–1^ for RO3280; 9.59 × 10^2^ M^–1^ for GSK461364). This fact confirms our previous hypothesis about site 1 as the preferential binding site into the protein. Synchronous fluorescence spectroscopy experiments were performed to analyze the changes in the microenvironment polarity around fluorescent residues of HSA. When the value of the intervals (Δλ) between the excitation and emission wavelengths is set at 15 nm, synchronous fluorescence spectra offer characteristic information on tyrosine residues.^61−63^ As shown in Figure 8a,c, no significant shifts were observed in the fluorescence emission maximum after successive additions of RO3280 and GSK461364, suggesting that there are no substantial changes in the polarity of the microenvironment around the tyrosine residues. Figure 8b,d shows the synchronous fluorescence spectra of HSA (in the absence and presence of both inhibitors) for Δλ = 60 nm. The addition of RO3280 to the HSA solution leads to a red shift of the emission maximum of about 8 nm (for a [drug]:[protein] ratio of 7:1) (Figure 8b). This type of spectral shift is typically attributed to an increase in the polarity of the microenvironment around the single tryptophan residue located in binding site I.^61−63^ In the case of Figure 8d, the evolution of the emission band of HSA upon titration with GSK461364 cannot be clearly observed due to the distortion caused by the fluorescence emission band of GSK461364.

**Figure 9:**
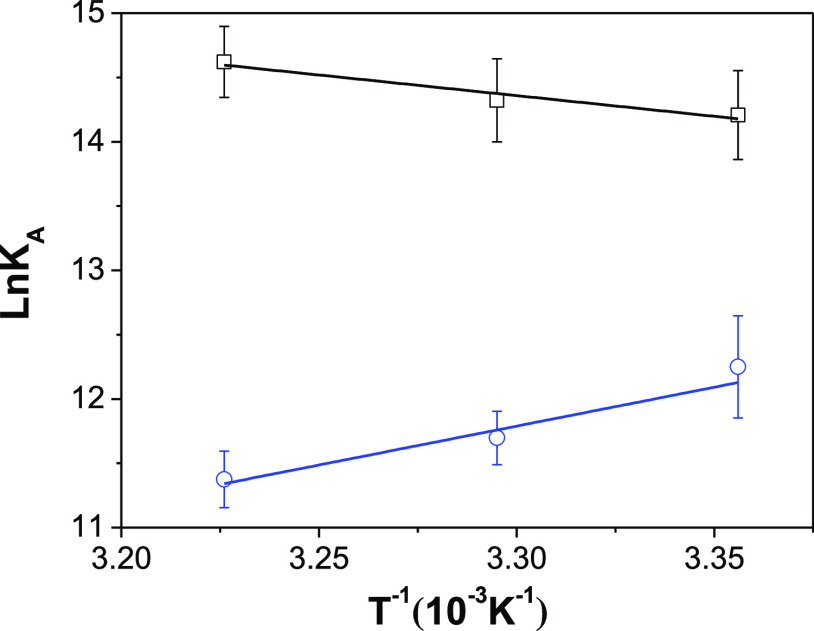
Van’t Hoff plot for the binding of RO3280 (black squares) and GSK461364 (blue circles) to HSA. Error bars correspond to ±σ.

The dependence of the binding constants of GSK461364 and RO3280 with temperature were also investigated to obtain the thermodynamic parameters of the formation of the HSA–drug complexes. Enthalpy (Δ*H*) and entropy (Δ*S*) changes were obtained from the van′t Hoff equation
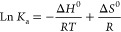
6and free energy (Δ*G*) changes were calculated from
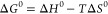
7The van’t Hoff plots are shown in Figure 9, and the corresponding thermodynamic parameters are collected in Table 2. A comparison between the thermodynamic parameters determined for the complex formation of RO3280 and GSK461364 with respect to some kinase inhibitors is shown in Table 3. The binding process of GSK461364 with HSA is exothermic as for most of the inhibitors. The negative enthalpy and entropy determined for GSK461364 suggests the implication of hydrogen bonds and van der Waals interactions in the formation of the protein–drug complex.^64^ On the other hand, the binding process of RO3280 is endothermic and entropy-driven as previously reported for BI-2536.^19^ Positive enthalpies are typically attributed to hydrophobic interaction but, in the cases of RO3280 and BI-2536, it could be associated with the proton pre-equilibrium described before.^65^ Proton-releasing processes in protein–ligand interactions generally result in a positive enthalpy.^64^ Thus, the enthalpies determined for the binding processes of both drugs to HSA could involve two equilibrium stages, i.e., the proton pre-equilibrium and the binding to the hydrophobic pocket.^65^

## 4 Conclusions

The spectroscopic properties and binding parameters to HSA of the PLK1 inhibitors RO3280 and GSK461364 have been studied here. Both drugs showed fluorescence emission in organic solvents, and the emission spectrum of RO3280 is sensitive to the polarity of the medium. In aqueous solutions at the physiological pH, the fluorescence emission was only observed for GSK461364 (which also disappears at strong acidic conditions). We found that the charge states of RO3280 and GSK461364 are +2 and +1, respectively, at pH 7.4. The first protonation equilibrium occurs in the auxophore group of both drugs (piperidine and piperazine) without a significant impact on their electronic properties. On the contrary, the fluorescence emission disappears, and absorption spectra are strongly modified, when a proton is accepted by an amine group situated in the π-conjugated part of the molecule (second protonation equilibrium). The fluorescence emission of RO3280 reappears in the presence of HSA, and this fact was attributed to a deprotonation pre-equilibrium prior to the binding process to the protein. Competitive experiments with warfarin and ibuprofen indicated that both RO3280 and GSK461364 bind to site I of HSA. The results obtained in the synchronous fluorescence experiments suggested a change in the polarity of the microenvironment around the single tryptophan residue upon binding of RO3280 at site I. The binding constant of RO3280 (2.23 × 10^6^ M^–1^) is unusually high in relation to the constants reported for other kinase inhibitors. The binding processes of RO3280 and GSK461364 to HSA are entropy- and enthalpy-driven, respectively. The positive enthalpy found for the RO3280-HSA complex formation was associated with the deprotonation pre-equilibrium of RO3280 commented before.

## Supporting Information Available

Spectroscopic characterization; DFT calculations; and protein binding studies (PDF)
