# Supplementary material for: Unusually High Affinity of the PLK Inhibitors RO3280 and GSK461364 to HSA and Its Possible Pharmacokinetic Implications
Source: Mol Pharm. 2023 Feb 22;20(3):1631–42. doi: 10.1021/acs.molpharmaceut.2c00849 (PMC9997069; doi:10.1021/acs.molpharmaceut.2c00849)
Supplement: Supplementary file 1 — mp2c00849_si_001.pdf [file mp2c00849_si_001.pdf]

Unusually high affinity of the PLK inhibitors RO3280 and GSK461364 to HSA and its possible pharmacokinetic implications

Jesús Fernández-Sainz,[a] Pedro J. Pacheco-Liñán,[a] Consuelo Ripoll,[a] Joaquín González-Fuentes,[b] José Albaladejo,[c] Iván Bravo,[a,b] and Andrés Garzón-Ruiz [a,*]

[a] *Departamento de Química Física, Facultad de Farmacia, Universidad de Castilla-La Mancha, Av. Dr. José María Sánchez Ibáñez, s/n, 02071 Albacete, Spain.*

[b] *Centro Regional de Investigaciones Biomédicas (CRIB), Unidad Asociada de Biomedicina (UCLM-CSIC), C/ Almansa, 14, 02008 Albacete, Spain*

[c] *Departamento de Química Física, Facultad de Ciencias y Tecnologías Químicas, Universidad de Castilla-La Mancha, Avenida Camilo José Cela, 10, 13071 Ciudad Real, Spain.*

Supporting Information

**Table of contents**	**Page**
S1. Spectroscopic characterization	**S2**
S2. DFT calculations	**S5**
S3. Protein binding studies	**S7**

S1. Spectroscopic characterization

Table S1. Maximum absorption and emission wavelengths (λ_{ab}^{max} and λ_{em}^{max}) of RO3280 and GSK461364 in different solvents.

	RO3280		GSK461364	
**Solvent**	**λ_{ab}^{max} (nm)**	**λ_{em}^{max} (nm)**	**λ_{ab}^{max} (nm)**	**λ_{em}^{max} (nm)**
Dichloromethane	327	429	309	376
Ethanol	326	385	306	375
Acetonitrile	325	462	307	374
DMSO	329	451	309	375
Water (pH 7.0)	321	–[a]	306	380
Water (pH 13.0)	322	452[a]	–	–

[a] Very weak fluorescence signal at pH 7.0

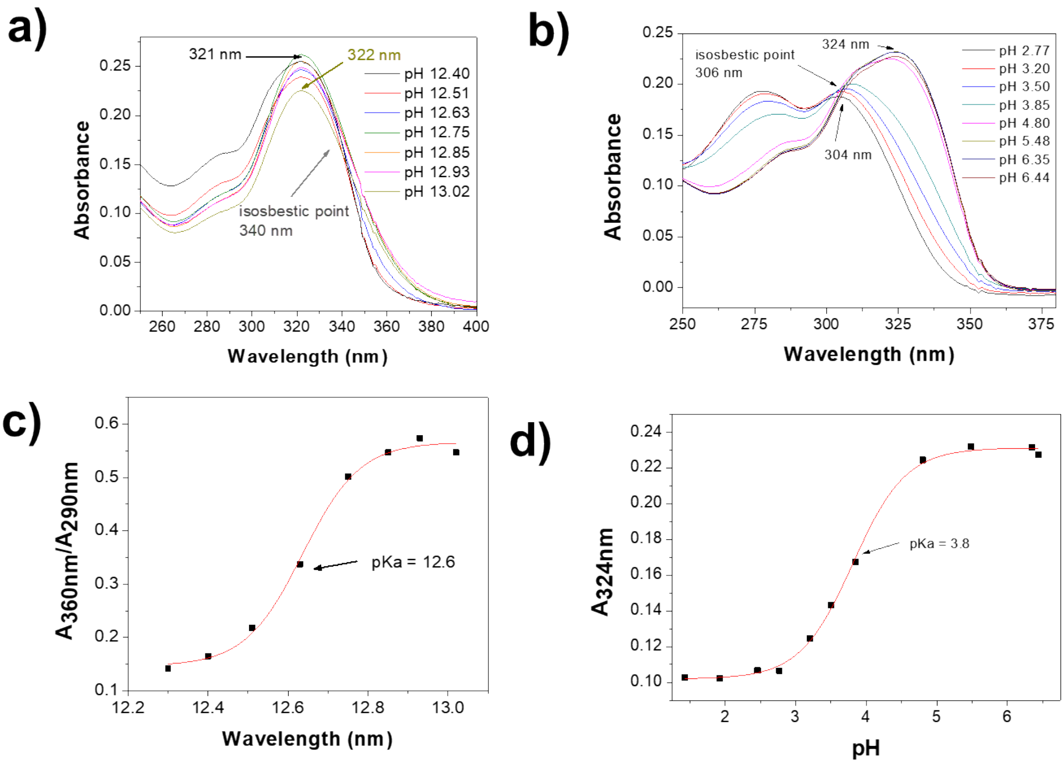

Figure S1. (a) UV-Vis absorption spectrum of RO3280 recorded at different pH values (in the basic range). (b) UV-Vis absorption spectrum of RO3280 recorded at different pH values (in the acid range). (c) Absorbance measured at 360 and 290 nm vs. pH (in the basic range). (d) Absorbance measured at 324 nm vs. pH (in the acid range)

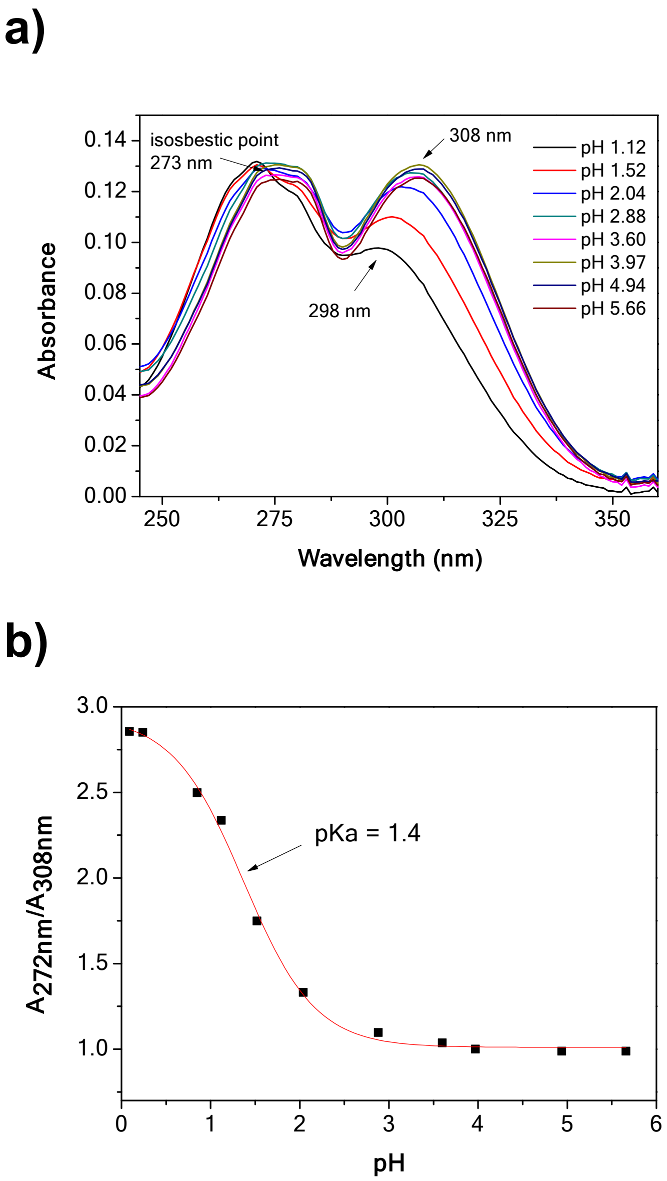

Figure S2. (a) UV-Vis absorption spectrum of GSK461364 recorded at different pH values. (b) Ratio of absorbance measured at 272 and 308 nm vs. pH

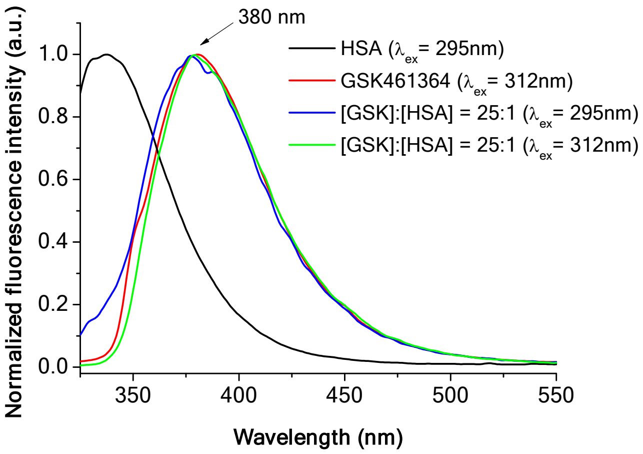

Figure S3. Fluorescence emission spectra of HSA, GSK461364 and a mixture of both compounds with a [GSK461364]:[HSA] ratio of 25:1. The emission spectrum of the mixture of HSA and GSK461364 was measured at different excitation wavelengths (λ_{ex}).

S2. DFT calculations

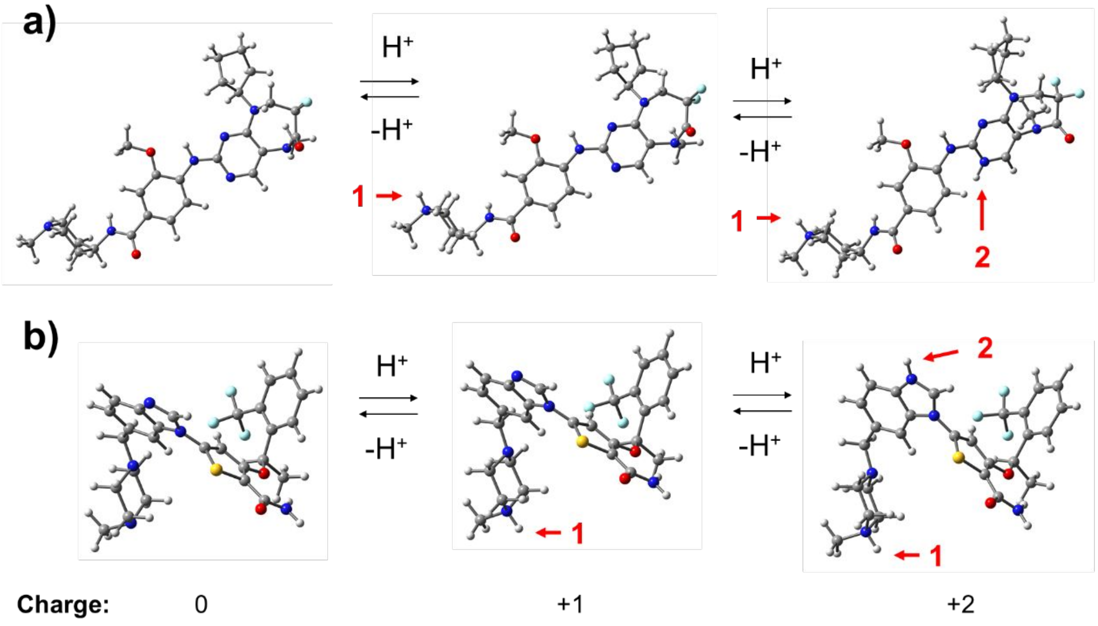

Figure S4. Protonation equilibria (and protonation sites) of (a) Ro3280 and (b) GSK461364

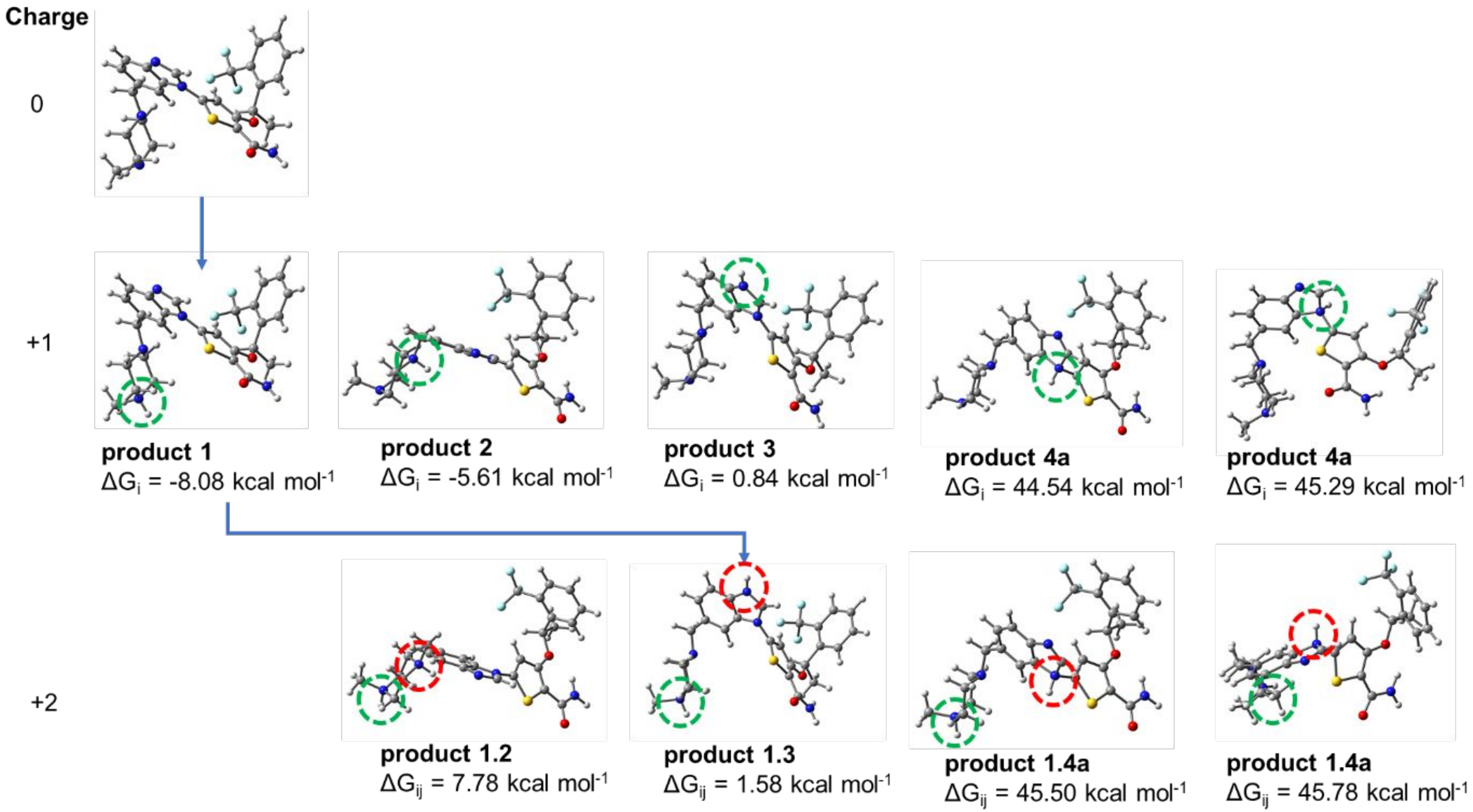

Figure S5. Optimized molecular geometry of GSK461364 and different protonated products. Products 1, 2, 3, 4a and 4b correspond to all the possible monoprotonated forms of GSK461364. Products 1.2, 1.3, 1.4a and 1.4b correspond to all the possible diprotonated forms starting from the most energetically favorable monoprotonated form (product 1). ΔG_i and ΔG_{ij} are the Gibbs free energies calculated for the first and second protonation equilibria. The dotted circles indicate the ionized groups.

S3. Protein binding studies

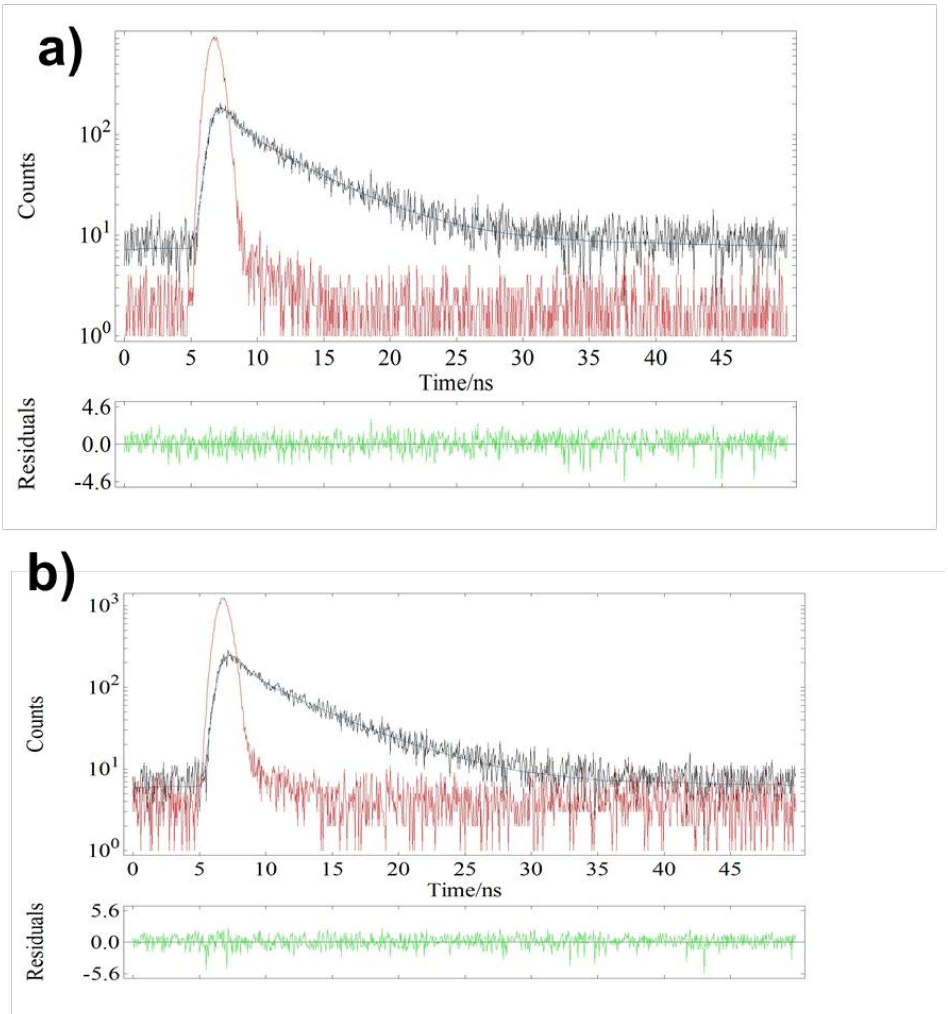

Figure S6. Fluorescence intensity decay of HSA (1 μM) in the presence of (a) GSK461364 and (b) Ro3280 in a [drug]:[HSA] ratio of 3:1 at 298 K.

Table S2. Fluorescence decay data measured for HSA in the absence and presence of drug (298 K).

**[Ro-3280]:[HSA]**	**τ_1 (ns)**	**τ_2 (ns)**	**τ_3 (ns)**	**$\alpha_1\times10^{-3}$**	**$\alpha_2\times10^{-3}$**	**$\alpha_3\times10^{-3}$**	**χ^2**	**τ_m (ns)**	**$\tau_{m,0}/\tau_m$**
0:1	0.06271	1.816	5.966	124.1	9.935	13.75	1.047	4.846	
1:1	0.4906	2.780	6.408	14.68	7.713	8.631	1.124	4.974	0.974
2:1	0.6993	4.605	8.841	12.09	10.21	1.332	1.300	4.856	0.998
3:1	0.5295	3.937	8.157	9.676	7.506	2.456	1.176	5.163	0.939
**[GSK461364]:[HSA]**	**τ_1 (ns)**	**τ_2 (ns)**	**τ_3 (ns)**	**$\alpha_1\times10^{-3}$**	**$\alpha_2\times10^{-3}$**	**$\alpha_3\times10^{-3}$**	**χ^2**	**τ_m (ns)**	**$\tau_{m,0}/\tau_m$**
0:1	0.1581	2.225	6.289	33.59	8.725	9.412	1.233	4.961	
1:1	0.4356	3.139	6.531	13.46	6.966	6.960	1.085	5.029	0.986
2:1	0.06798	1.553	5.834	54.13	6.060	8.068	1.131	4.812	1.031
3:1	0.5618	3.095	7.015	8.844	5.477	4.291	1.214	5.121	0.969

Table S3. Binding parameters corresponding to the formation of HSA-drug complex at different temperatures. Data obtained including inner filter conrrections are indicated with the superscript "c". The superscript "u" is used for data without inner filter conrrections.

**GSK-461364**				
**T (K)**	**$n^c \pm 2\sigma$**	**$\log K_a^c \pm 2\sigma$**	**$n^u \pm 2\sigma$**	**$\log K_a^u \pm 2\sigma$**
310	1.11 ± 0.05	4.94 ± 0.19	1.23 ± 0.06	5.75 ± 0.24
303	1.15 ± 0.03	5.08 ± 0.18	1.28 ± 0.05	5.97 ± 0.24
298	1.19 ± 0.06	5.32 ± 0.35	1.29 ± 0.10	6.06 ± 0.54
**Ro3280**				
**T (K)**	**$n^c \pm 2\sigma$**	**$\log K_a^c \pm 2\sigma$**	**$n^u \pm 2\sigma$**	**$\log K_a^u \pm 2\sigma$**
310	1.33± 0.05	6.35 ± 0.24	1.52 ± 0.07	7.55 ± 0.39
303	1.30 ± 0.07	6.22 ± 0.27	1.50 ± 0.08	7.49 ± 0.36
298	1.29 ± 0.06	6.17 ± 0.30	1.62 ± 0.11	8.04 ± 0.58
